# Psychiatric Symptoms of Patients With Anti-NMDA Receptor Encephalitis

**DOI:** 10.3389/fneur.2019.01330

**Published:** 2020-01-24

**Authors:** Wei Wang, Le Zhang, Xiao-Sa Chi, Li He, Dong Zhou, Jin-Mei Li

**Affiliations:** ^1^Department of Neurology, West China Hospital, Sichuan University, Chengdu, China; ^2^Department of Neurology, Xuanwu Hospital, Capital Medical University, Beijing, China

**Keywords:** anti-NMDA receptor encephalitis, psychiatric symptoms, antibody titer, treatment strategy, neurological symptoms

## Abstract

**Objective:** We conducted this study to analyze the clinical characteristics of the psychiatric symptoms of patients with anti-NMDAR encephalitis.

**Methods:** A retrospective study of anti-NMDAR encephalitis in China was performed. The clinical characteristics of the psychiatric symptoms, the relationship between the antibodies titers and clinical characteristics of patients with anti-NMDAR encephalitis were determined.

**Results:** A total of 108 patients with a definitive diagnosis of anti-NMDAR encephalitis were included in this study. 103 patients (95%) developed one or several psychiatric symptoms. The comparison of the high titer group and the low titer group showed that more patients presented psychiatric symptoms as the initial symptom in the high titer group (*P* = 0.020), the prevalence of the symptoms such as depressive, catatonic, and central hypoventilation were also higher in the high titer group than the low titer group (*P* = 0.033, 0.031 and 0.006, respectively). Meanwhile, more patients received a combination treatment of IVIg and corticosteroids in the high titer group than the low titer group and patients in high titer group were prescript with anti-psychiatric drugs more often than the patients in low titer group (*P* = 0.026 and 0.003, respectively).

**Conclusions:** Psychiatric symptoms are the most common clinical characteristics of patients with anti-NMDAR encephalitis. Patients with higher antibodies titers more often presented with psychiatric symptoms as the initial symptom, and showed a more severe clinical feature. Screening for the anti-NMDAR antibodies is essentially important in patients who present psychiatric symptoms with or without other neurological symptoms.

Anti–N-methyl-D-aspartate receptor (NMDAR) encephalitis is an autoimmune disorder in which IgG antibodies are directed against the NR1 subunit of the NMDAR. The disorder includes a range of psychiatric symptoms early in the course of the disease, followed by neurological symptoms, such as memory problems, seizure, decreased consciousness, dyskinesia, autonomic instability and hypoventilation (1, 2). The psychiatric symptoms of this disorder could present as psychosis, anxiety, insomnia, mania, and catatonic symptoms (3, 4), and many patients were initially seen by psychiatrists or admitted to psychiatric centers. As anti–NMDAR encephalitis is an IgG antibody mediated autoimmune disorder, the severity of the disease is closely related to the titers of the antibodies. Previous studies have shown that patients who improved had a parallel decrease of serum titers, whereas those who did not improve maintained high titers in serum and cerebrospinal fluid (CSF) (2). Although immunotherapies have been suggested to be the first-line treatment strategy of patients with anti-NMDAR encephalitis, and 79% patients can reach a good outcome (3), anti-psychotic therapies may still be implanted due to the prominent psychiatric symptoms. Many of the studies to date that have looked at treatment algorithms for anti-NMDR encephalitis have focused on immunotherapy, with only a few reports looking at treatment of the psychiatric manifestations of the disease. Therefore, we conducted this study to further analysis the clinical characteristics of the psychiatric symptoms of patients with anti-NMDAR encephalitis, as well as the relationship between the antibodies titers and clinical characteristics, especially psychiatric symptoms, and the treatment strategy of patients with anti-NMDAR encephalitis.

## Methods

### Patients

Patients who presented with psychiatric symptoms, seizures or focal neurological signs were tested for the presence of NMDAR antibodies in serum or CSF sample at the West China Hospital of Sichuan University between June 2011 and April 2016. All patient who tested positive for anti-NMDAR antibodies in CSF or/and serum were included in this study and patients lacking key clinical data or suspecting of virus encephalitis or other infectious encephalitis were excluded. The diagnosis of anti-NMDAR encephalitis is consistent with previous studies (5, 6). Clinical information was obtained by the authors or referring physicians at symptom onset and at regular intervals during the course of the disease. Fifty-one patients have been partly reported (5). We systemically reviewed the psychiatric symptoms of each patient in the following category: aggression, depressive, catatonic, disorganized, anxious, psychotic (hallucination or delusions), manic, suicidal, and insomnia. The diagnosis of each category meet the *Diagnostic and Statistical Manual of Mental Disorders*, Fifth Edition (DSM-5) (7, 8).

The outcome was assessed using the modified Rankin Scale (mRS) at the last visit (9, 10). A diagnosis of anti-NMDAR encephalitis was considered to be definitive when (1) encephalitic signs such as psychiatric symptoms, seizures, or focal neurological signs were shown; (2) anti-NMDAR antibodies were detected in CSF or/and serum. Central hypoventilation was considered if the patient needs respiratory support such as mechanical ventilation. Relapse was defined as the onset or worsening of symptoms at least 2 months after improvement.

### Subgroup Analysis

Based on the titer of the antibodies in the serum and CSF, we divided all the patients into two groups: low titer group and high titer group. The psychiatric symptoms were compared between these two groups. The antibody titers ≧1:32 in CSF / ≧1:100 in serum were considered high titer, and the antibody titer <1:32 in CSF/ <1:100 in serum were considered low titer.

### Antibodies Study

This study was approved by the Research Ethics Committee of Sichuan University. Written informed consent was obtained from each subject. The serum and /or CSF samples of all patients were sent to two institutions (Oumeng Biotechnology Corporation, or Peking Union Medical College Hospital, Beijing, China) to detect antibodies against NMDAR, contactin-associated protein 2 (CASPR2), GABAR B1/B2, AMPA receptors 1/2 and leucine-rich glioma-inactivated protein 1 (LGI1). The data that support the findings of this study are available from the corresponding author upon reasonable request. Samples were classified as positive or negative by indirect immunofluorescence using EU 90 cells according to previous study (5).

### Statistical Analyses

Statistical analyses were performed using SPSS version 20.0. We performed a univariate analysis in continuous variables such as age. Gender and different clinical symptoms were analyzed as categorical variables. The independent *t*-test or one-way analysis of variance (ANOVA) was used for continuous variables, and the Chi-square test or Fisher's exact test was used for categorical variables. When counts of zero cells were recorded, odds ratios (ORs) were calculated using Haldane's modification, which adds 0.5 to all counts to accommodate possible zero counts (11). *P*-values <0.05 (two-sided) were considered to be significant.

## Results

A total of 108 patients with a definitive diagnosis of anti-NMDAR encephalitis were included in this study. Seventy-eight patients had positive antibody results in both CSF and serum, and 30 patients only had CSF positive antibody results. The test for other autoimmune antibodies, such as antibodies against AMPA receptors 1/2, CASPR2, LGI1, and GABAR B1/B2 were negative. The demographic and general clinical characteristics are summarized in Table 1. Sixty-three patients were female (58%), and the mean age was 27.1 years (range: 9–71).

**Table 1:** Clinical characteristics of patients with anti-NMDAR encephalitis.

**Clinical features**	**Patients (%)**
Number	108 (100%)
Female	63 (58%)
Median age, range (years)	27.1, 9–71
Prodromal symptoms	53 (49%)
Initial symptoms	–
Psychiatric	62 (57%)
Neurological	42 (39%)
Unspecific	4 (4%)
Psychiatric symptoms	103 (95%)
Aggression	43 (40%)
Depressive	28 (26%)
Catatonic	15 (14%)
Disorganized	76 (70%)
Anxious	26 (24%)
Psychotic	54 (50%)
Manic	67 (62%)
Suicidal	11 (10%)
Insomnia	28 (26%)
Neurological symptoms	102 (94%)
Seizure	89 (82%)
Memory deficits	56 (52%)
Speech disturbances	68 (63%)
Dyskinesias and movement disorders	47 (44%)
Autonomic instability	45 (42%)
Decreased consciousness	70 (65%)
Central hypoventilation	27 (25%)
Abnormal MRI findings^a^	50 (48%)
Abnormal EEG findings^b^	71 (74%)
Abnormal CSF findings^c^	76 (70%)
Tumor	15 (14%)

a *11 patients with increased signal on T2-weighted or fluid-attenuated inversion recovery (FLAIR) images of the medial temporal lobe; six with contrast enhancement of the cerebral cortex; four with contrast enhancement of meninges; one with contrast enhancement of the temporal lobes and basal ganglia; eight with multifocal cortical and subcortical changes; 15 with multifocal white-matter changes; four with cortical atrophy; and one with empty sella*.

b *47 patients (66%) with bilateral or unilateral generalized slow waves without epileptiform discharges and 24 patients (34%) with epileptiform discharges*.

c *70 patients (92%) with pleocytosis and 35 patients (46%) with increased protein concentrations*.

### Clinical Symptoms

Sixty-two patients (57%) presented with psychiatric symptoms as the initial symptom, including mood alteration (anxious or depressive), aggression, manic, delusion, and visual or auditory hallucinations. Forty-two patients (39%) presented with neurological symptoms, such as seizure, movement disorder, and speech disturbances, as the initial symptom.

During the disease course, 103 patients (95%) developed one or several psychiatric symptoms, including aggression (43 patients, 40%), depressive (28 patients, 26%), catatonic (15 patients, 14%), disorganized (76 patients, 70%), anxious (26 patients, 24%), psychotic (54 patients, 50%), manic (67 patients, 62%), suicidal (11 patients, 10%) and insomnia (28 patients, 26%). Hundred and two patients (94%) in this cohort had neurological symptoms, including seizures in 89 patients (82%), memory deficits in 56 patients (52%), speech disturbances in 68 patients (63%), dyskinesias and movement disorders in 47 patients (44%), autonomic instability in 45 patients (42%), decreased consciousness in 70 patients (65%) and central hypoventilation in 27 patients (25%).

### Subgroup Analysis—Low Titer Group Verses High Titer Group

**Figure 1:**
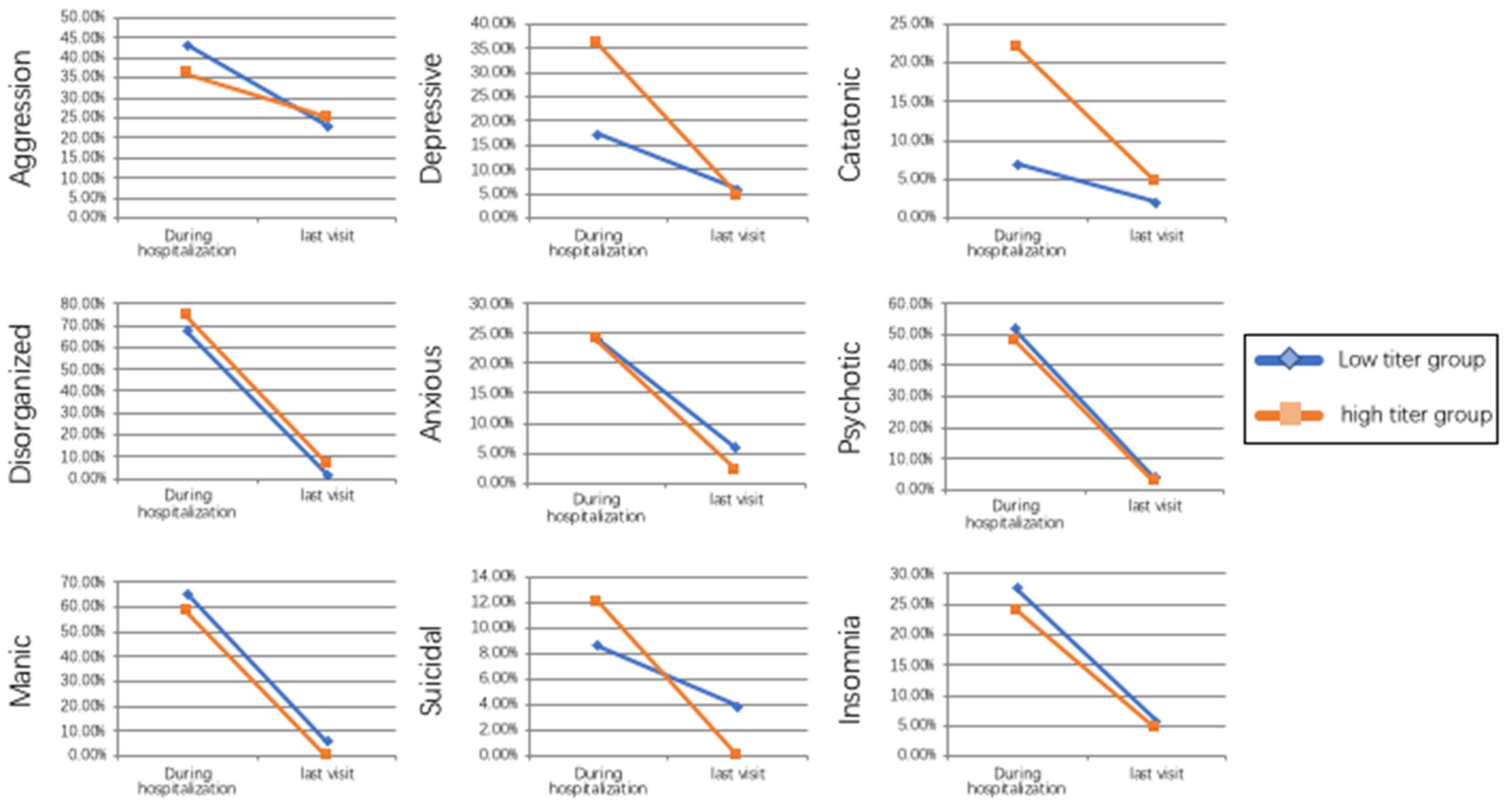
Tendency of the frequency of different psychiatric symptoms between the low titer group and high titer group during the hospitalization and at last visit.

The results of the comparison between the low titer group and high titer group shown in Table 2 and Figure 1. In total, 58 patients were included in the low titer group and 50 patients were included in the high titer group. Twenty-seven patients presented psychiatric symptoms as the initial symptom in the low titer group while 35 patients in the high titer group presented psychiatric symptoms as the initial symptom (*P* = 0.020, OR = 0.373, 95% CI: 0.169–0.827). Four patients had catatonic symptoms in the low titer group while 11 patients had catatonic symptoms in the high titer group (*P* = 0.031, OR = 0.263, 95% CI: 0.078–0.886). Ten patients experienced depressive in the low titer group whereas 18 patients experienced depressive in the high titer group (*P* = 0.033, OR = 0.370, 95% CI: 0.152–0.905). Eight patients in the low titer group whereas 19 patients in the high titer group suffered from central hypoventilation (*P* = 0.006, OR = 0.261, 95% CI: 0.102–0.668), and eight patients in the low titer group and 18 patients in the high titer group had mechanical ventilation during the period of hospitalization (*P* = 0.011, OR = 0.284, 95% CI: 0.111–0.731). The frequency of other psychiatric symptoms and clinical features, such as aggression, disorganized, anxious, psychotic, manic, suicidal, insomnia, prodromal symptoms, seizure, memory deficits, speech disturbances, dyskinesia and movement disorders, autonomic instability, decrease consciousness, tracheotomy, abnormal MRI findings, abnormal EEG findings, abnormal CSF findings, and the tumor presentation rate were not significantly different between the two groups. We also compared the treatment strategies between the high titer group and low titer group. And we found that patients in high titer group were prescript with anti-psychiatric drugs more often than the patients in low titer group (*P* = 0.003, OR = 0.168, 95% CI: 0.046–0.616), but the frequencies of different types of anti-psychiatric drugs were not significantly different. Meanwhile, more patients received a combination treatment of IVIg and corticosteroids in the high titer group than the low titer group (*P* = 0.026, OR = 0.415, 95% CI: 0.190-0.906). In addition, the relapse rate, the mean mRS and the frequency of the residual symptoms at the last visit were not shown significant difference between the two groups.

**Table 2:** Comparison between patients with low antibody titer and high antibody titer.

	**Low titer group (*n* = 58)**	**High titer group (*n* = 50)**	***P***	**OR**	**95% CI**
Mean age	26.17 ± 11.70	28.26 ± 11.75	0.421	–	–
Female: male	34: 24	29: 21	1.000	1.026	0.476–2.209
Mean hospital stay/months	33.40 ± 26.80	36.56 ± 23.36	0.944	–	–
Psychiatric symptom as initial symptom	27	35	0.020	0.373	0.169–0.827
Aggression	25	18	0.557	1.347	0.619–2.929
Depressive	10	18	0.033	0.370	0.152–0.905
Catatonic	4	11	0.031	0.263	0.078–0.886
Disorganized	39	37	0.531	0.721	0.312–1.665
Anxious	14	12	1.000	1.008	0.416–2.441
Psychotic	30	24	0.844	1.161	0.544–2.474
Manic	38	29	0.430	1.376	0.631–3.002
Suicidal	5	6	0.749	0.692	0.198–2.420
Insomnia	16	12	0.828	1.206	0.507–2.873
Prodromal symptom	32	21	0.182	1.700	0.792–3.648
Seizure	46	43	0.462	0.624	0.225–1.732
Memory deficits	29	27	0.700	0.852	0.399–1.817
Speech disturbances	35	33	0.552	0.784	0.357–1.722
Dyskinesias and movement disorders	21	26	0.119	0.524	0.242–1.133
Autonomic instability	25	20	0.844	1.136	0.527–2.450
Decreased consciousness	34	36	0.168	0.551	0.245–1.237
Central hypoventilation	8	19	0.006	0.261	0.102–0.668
Mechanical ventilation	8	18	0.011	0.284	0.111–0.731
Tracheotomy	6	9	0.280	0.526	0.173–1.597
Abnormal MRI findings	26 (*n* = 56)	26 (*n* = 48)	0.554	0.733	0.338–1.589
Abnormal EEG findings	39 (*n* = 51)	25 (*n* = 35)	0.627	1.300	0.489–3.457
Abnormal CSF findings	40	38	0.519	0.702	0.299–1.650
Tumor	7	8	0.595	0.721	0.241–2.151
Results of follow-up					
Total number	52	44	–	–	–
Relapse	3	4	0.698	0.612	0.129–2.896
Mean mRS	1.12 ± 1.778	1.52 ± 1.886	0.477	–	–
Sequlae	–	–	–	–	–
Memory deficits	6	11	0.110	0.391	0.131–1.165
Speech disturbances	6	1	0.120	5.609	0.648–48.509
Seizure	2	0	0.498	2.647	0.266–26.360
Psychiatric symptoms	–	–	–	–	–
Aggression	12	11	1.000	0.900	0.352–2.302
Depressive	3	2	1.000	1.286	0.205–8.064
Catatonic	1	2	0.592	0.412	0.036–4.700
Disorganized	1	3	0.324	0.268	0.027–2.673
Anxious	3	1	0.632	2.633	0.264–26.257
Psychotic	2	1	1.000	1.720	0.151–19.632
Manic	3	0	0.253	3.600	0.388–33.413
Suicidal	2	0	0.495	2.647	0.266–26.360
Insomnia	3	2	1.000	1.286	0.205–8.064
Treatment strategy	–	–	–	–	–
Immunotherapy	55	50	0.370	0.275	0.030–2.537
IVIg monotherapy	28	21	0.514	1.289	0.602–2.762
Corticosteroids monotherapy	5	2	0.447	2.264	0.420–12.218
Combination of IVIg and corticosteroids	19	27	0.026	0.415	0.190–0.906
Combination of IVIg, corticosteroids and plasma exchange	1	0	1.000	1.759	0.155–19.971
Combination of IVIg, corticosteroids and second-line therapy	2	0	0.622	2.684	0.271–26.626
Anti-psychiatric drugs	42	47	0.003	0.168	0.046–0.616
Anti-psychotic drugs	18	19	0.543	0.734	0.331–1.629
Anti-anxiety drugs	2	2	1.000	0.857	0.116–6.318
Combination of anti-psychotic drugs and anti-anxiety drugs	17	21	0.225	0.573	0.258–1.270
Combination of anti-psychotic and anti-depressive drugs	1	0	1.000	1.759	0.155–19.971
Combination of anti-psychotic, anti-anxiety, and anti-depressive drugs	4	5	0.730	0.667	0.169–2.631
Tumor resection	6	6	0.785	0.846	0.255–2.811

*IVIg, intravenous immunoglobulin*.

### Treatment Strategies

As for the treatment strategies, 105 out of 108 patients (97%) received immunotherapy, including 49 patients treated with intravenous immunoglobulin (IVIg, 0.4 g/kg per day for 5 days) once or several times, seven patients were treated with intravenous methylprednisolone (1 g/day for 5 days) alone, 46 patients received a combination treatment of IVIg and intravenous methylprednisolone, one patient received a combination treatment of IVIg, intravenous methylprednisolone and plasma exchange, and two patients received IVIg, intravenous methylprednisolone and a second-line therapy (one with cyclophosphamide, one with rituximab). Eighty-nine patients (82%) were prescript drugs to control the psychiatric symptoms, including 37 patients with anti-psychotic drugs alone, four patients with anti-anxiety drugs alone, 38 patients with a combination of anti-psychotic drugs and anti-anxiety drugs, one patient with a combination of anti-psychotic and anti-depressive drugs, 9 patients with a combination of anti-psychotic, anti-anxiety and anti-depressive drugs. The antipsychotic drugs include typical (haloperidol and tiapride) and atypical (risperidone, olanzapine, quetiapine, and clozapine). Three patients had electroconvulsive therapy (ECT) isolated or combined with other anti-psychotic drugs. Twelve patients underwent tumor resection.

As for the side effect of the antipsychotics, three patients had extrapyramidal symptoms, one patient had tardive dyskinesia, two patients had decreased consciousness (somnolence) and one patient had liver function damage after using antipsychotics. However, as all patients were prescript with other drugs at the same time, the side effects may also due to the use of other drugs and may also reflect the natural progression of anti-NMDA receptor encephalitis.

### Follow-Up

The median follow-up duration was 17 months (1–47 months). At the last visit, the mean mRS was 1.33 ± 1.83. Forty-four patients (46%) reached fully recovery (mRS = 0), 33patients (34%) had mild deficits (mRS 1-2), 10 patients (11%) had severe deficits (mRS 3-5), and nine patients (9%) died. During the follow-up period, 17 patients still had memory deficits, seven had speech disturbance, and two had one or several episodes of seizures. The remaining psychiatric symptoms included aggression (23 patients), depressive (five patients), catatonic (three patients), disorganized (four patients), anxious (four patients), psychotic symptoms (three patients), manic (three patients), suicidal (two patients) and insomnia (five patients). Seven patients relapsed during the course of this study. Twelve patients were lost to follow-up.

## Discussion

The present study not only further proved that Chinese patients with anti-NMDAR encephalitis might have a lower incidence of tumor and a relatively better outcome compared with studies from other countries and regions, which is consistent with our previous study (5), but also reveal several features mainly focus on the characteristic and treatment of the psychiatric symptoms of patients with anti-NMDAR encephalitis.

Psychiatric symptoms are the most common clinical characteristic of patients with anti-NMDAR encephalitis; the incidence is around 65–80% (2, 9, 12). Antibodies against NMDAR may be associated with psychiatric symptoms for several reasons. Studies have shown that dysfunction of NMDAR and the glutamatergic system may be associated with the pathogenesis of schizophrenia, as antagonists of NMDAR, including phencyclidine and ketamine, have been shown to induce psychotic symptoms (positive and negative) and behavioral and cognitive impairments similar to those observed in patients with schizophrenia, a finding which suggests that NMDAR hypofunction may lead to secondary dopaminergic dysregulation (13–15). Isolated psychiatric episodes can also be found in patients with positive anti-NMDAR antibodies, in a prevalence of ~4%, either at disease onset or relapse (16, 17). And patients with isolated psychiatric symptoms or milder presentations do not necessarily progress to more severe multi-symptom stage, despite prolonged periods without treatment (17).

Recently, several studies explored the relationship between anti-NMDAR antibodies and well-defined psychiatric disorders, such as schizophrenia, major depressive disorder, and borderline personality disorder (18–21). Although it is reported that several types of serum NMDAR antibodies were present in 9.9% of acutely ill patients who were initially diagnosed with schizophrenia (21), the antibody subtype profile of these patients differed from those of non-schizophrenic anti-NMDAR encephalitis patients, and the frequency of antibody positivity in these patients was similar to that observed in the controls (22–24). Kawai et al. reviewed a group of patients with mood disorder and found that four out of 13 patients whose CSF was obtained had positive anti-NMDAR antibodies. And all of the four patients developed some neurological symptoms during the clinical course (25). This study presented the relationship between psychiatric disorders (mood disorder) and anti-NMDA receptor antibodies from a different perspective. Positive anti-NMDA receptor antibodies in patients with psychiatric symptoms usually indicate neurological disease rather than purely psychiatric disorders, and the treatment strategy also need to be changed according to the underline etiology.

In the present study, we divided the patients into two groups according to the antibodies titers and found the patients with higher antibodies titers more often presented psychiatric symptoms as the initial symptom and showed a more severe clinical feature. More patients in the high titer group presented depressive and catatonic symptoms, and more patients had central hypoventilation which may need mechanical ventilation or tracheotomy. Previous studies have shown that depressive and catatonia are the most common psychiatric symptoms in patients with anti-NMDAR encephalitis (26). As anti–NMDAR encephalitis is an IgG antibody mediated autoimmune disorder, the severity of the disease is closely related to the titers of the antibodies. Patients who improved had a parallel decrease of serum titers, whereas those who did not improve maintained high titers in CSF and serum (2).

From clinical practice we noticed that patients with a higher titer might have a more severe clinical impression, especially in patients who presented catatonic/stupor and patients who need mechanical ventilation. Although catatonia has commonly been ascribed to schizophrenia, it is more commonly seen in affective disorders and medical and neurological disorders, and anti-NMDAR encephalitis is considered to be an autoimmune type of catatonia. (27). The prevalence of catatonia is estimated to be 10–25% in the mixed inpatient populations of psychiatric institutions, however, there is a lack of systematic investigations on the presentation of catatonia in different patient groups (28, 29). It has been proposed that catatonia results from dysregulation in the glutamate, GABA-A, and dopamine pathways (30), and the NMDA receptor antagonist ketamine elicited catatonia-like signs when administered in healthy subjects (31, 32). And hypoventilation can be a results of the disruption of NR1 subunit of the NMDA receptor (33).

Previous studies showed that anti-NMDAR encephalitis often has a good outcome if appropriate treatment such as immunotherapy or tumor removal are carried out in time (3), however, as high as 45% of our patients had residual symptoms such as memory deficits, speech disturbances and especially psychiatric symptoms, which may still affect the quality of life of these patients. It is suggested that around 25% of the patients with anti-NMDAR encephalitis are left with memory, cognitive, and motor deficits (34). The mild deficits symptoms such as poor attention, behavioral disinhibition, and sleep dysfunction during the recovery period may be due to the frontal-lobe dysfunction (2). Notably, although the frequency of the depressive and catatonic symptoms were significantly different due to the different antibodies titers in the early stage of the disease course, the frequency of the residual symptoms was no significant difference between the low titer group and high titer group, which suggests a relative good treatment results despite the former severe symptoms presented in the high titer group.

Although immunotherapies have been suggested to be the first-line treatment strategy of patients with anti-NMDAR encephalitis, and 79% patients can reach a good outcome (3), anti-psychotic therapies may still be implanted due to the prominent psychiatric symptoms, and a small portion of the patients only had psychiatric symptoms (17). Many of the studies to date that have looked at treatment algorithms for anti-NMDR encephalitis have focused on immunotherapy, with only a few reports looking at treatment of the psychiatric manifestations of the disease (27, 35). No specific guidelines exist for treatment of psychiatric symptoms in this setting. Our results showed that eighty-nine patients (82%) were prescript drugs in order to control the prominent psychiatric symptoms. Previous reports suggested that ECT may help stabilizing the psychiatric symptoms of patients with anti-NMDAR encephalitis (27). But some suggested that use of highly sedating medications such as quetiapine, chlorpromazine, valproic acid, and benzodiazepines; high-potency antipsychotics like haloperidol may exacerbate motor symptoms in patients with anti-NMDAR encephalitis (36). As all of our patients were prescript with anti-psychiatric drugs as an additional therapy to the immunotherapy, and the categories and the dosages of the drugs are too various, it is difficult to further confirm whether these anti-psychiatric drugs is truly effective or not. Further studies are needed to analyze the underlying mechanisms and effectiveness of the anti-psychiatric drugs in the setting of patients with anti-NMDAR encephalitis, and if effective, a standard treatment algorithm is also required.

There are some limitations in the present study. Given the retrospective nature of this study, there may have possible selection bias. All patients were recruited from the department of neurology, therefore, some of the patients who only presented with psychiatric symptoms may be underestimated and the evaluation of some psychiatric symptoms may be insufficient. And the prevalence of memory deficits may also be underestimated because patients with severe psychosis or cognitive disorders at the early stage of the disease cannot be evaluated. Meanwhile, the psychiatric symptoms were evaluated at the first admission and during the clinical course (when patients can cooperate) in the present study, so the evaluation time point of each patient may be different.

In conclusion, psychiatric symptoms are the most common clinical characteristics of patients with anti-NMDAR encephalitis. Patients with higher antibodies titers more often presented with psychiatric symptoms as the initial symptom, and showed a more severe clinical feature. Screening for the anti-NMDAR antibodies is essentially important in patients who present psychiatric symptoms with or without other neurological symptoms.

## Data Availability Statement

All datasets generated for this study are included in the article/supplementary material.

## Ethics Statement

The studies involving human participants were reviewed and approved by This study was approved by the Research Ethics Committee of Sichuan University. Written informed consent to participate in this study was provided by the participants' legal guardian/next of kin.

## Author Contributions

WW wrote the main manuscript, analyzed the data, made the Tables 1, 2, and Figure 1. WW, LZ, X-SC, and LH collected the data. DZ and J-ML came up with the main ideas, lead the research group, and arranged the work of all authors. All authors reviewed the manuscript.

### Conflict of Interest

The authors declare that the research was conducted in the absence of any commercial or financial relationships that could be construed as a potential conflict of interest.
